# The prediction of cancer-specific mortality in T1 non-muscle-invasive bladder cancer: comparison of logistic regression and artificial neural network: a SEER population-based study

**DOI:** 10.1007/s11255-023-03655-5

**Published:** 2023-06-06

**Authors:** Aleksander Ślusarczyk, Piotr Zapała, Zofia Olszewska-Ślusarczyk, Piotr Radziszewski

**Affiliations:** 1 Department of General, Oncological and Functional Urology, Medical University of Warsaw, Lindleya 4, 02-005 Warsaw, Poland; 2 Faculty of Electronics and Information Technology, Warsaw University of Technology, Warsaw, Poland

**Keywords:** Non-muscle-invasive bladder cancer, T1 stage, Survival, Artificial neural network, Deep learning

## Abstract

**Purpose:**

To identify the risk factors for 5-year cancer-specific (CSS) and overall survival (OS) and to compare the accuracy of logistic regression (LR) and artificial neural network (ANN) in the prediction of survival outcomes in T1 non-muscle-invasive bladder cancer.

**Methods:**

This is a population-based analysis using the Surveillance, Epidemiology, and End Results database. Patients with T1 bladder cancer (BC) who underwent transurethral resection of the tumour (TURBT) between 2004 and 2015 were included in the analysis. The predictive abilities of LR and ANN were compared.

**Results:**

Overall 32,060 patients with T1 BC were randomly assigned to training and validation cohorts in the proportion of 70:30. There were 5691 (17.75%) cancer-specific deaths and 18,485 (57.7%) all-cause deaths within a median of 116 months of follow-up (IQR 80–153). Multivariable analysis with LR revealed that age, race, tumour grade, histology variant, the primary character, location and size of the tumour, marital status, and annual income constitute independent risk factors for CSS. In the validation cohort, LR and ANN yielded 79.5% and 79.4% accuracy in 5-year CSS prediction respectively. The area under the ROC curve for CSS predictions reached 73.4% and 72.5% for LR and ANN respectively.

**Conclusions:**

Available risk factors might be useful to estimate the risk of CSS and OS and thus facilitate optimal treatment choice. The accuracy of survival prediction is still moderate. T1 BC with adverse features requires more aggressive treatment after initial TURBT.

**Supplementary Information:**

The online version contains supplementary material available at 10.1007/s11255-023-03655-5.

## Introduction

Bladder cancer (BC) is the tenth most common cancer worldwide with a significant morbidity burden associated with high recurrence and progression rates [1]. The majority of bladder tumours are diagnosed early—at the stage of non-muscle-invasive disease (NMIBC) [2]. Among NMIBC, T1 tumours which are recognized when cancer infiltrates the submucosa, constitute the most aggressive type, associated with a high progression risk and non-negligible mortality [3, 4]. T1 BC is an insidious disease, which might shortly progress to the muscle-invasive stage (up to 20% of patients progress) and the 5-year cancer-specific mortality (CSM) approximates 10% [3]. Transurethral resection of the bladder tumour (TURBT) with adjuvant intravesical Bacillus Calmette Guerin (BCG) therapy remains a standard treatment for T1 tumours and is most commonly chosen [2]. Such therapeutic approach can be insufficient to provide long-term disease-free survival [5]. Thus, definitive surgery (radical cystectomy) should be considered in T1 bladder cancer as it is characterized by higher efficacy in terms of disease eradication [2]. However non-negligible complication rate and morbidity constitute a major disadvantage of radical cystectomy (RC), thus it is still rarely chosen as a treatment option for T1 NMIBC [6]. Moreover, RC might be considered overtreatment in the majority of cases [7, 8]. On the other hand, deferred RC after NMIBC progression to muscle-invasive disease (MIBC) is associated with compromised survival compared to early RC in high-risk NMIBC [9, 10].

European Organisation for Research and Treatment of Cancer (EORTC) and Club Urologico Español de Tratamiento Oncologico (CUETO) scoring models are well-established tools for the estimation of recurrence- and progression-free survival in NMIBC [4, 11]. Currently used nomograms are based on machine learning techniques with Cox proportional hazards (CPH) [4, 11, 12]. Above tools provide moderate accuracy and new approaches to predict the disease course are awaited [2]. This includes the pursuit of novel molecular markers and new risk factors which affect the course of the disease, but also the implementation of novel prediction tools [5]. Moreover, overall- (OS) and cancer-specific survival (CSS) are not frequently studied endpoints in NMIBC and above scoring systems were not designed to be used in that setting [4, 11, 12].

Artificial intelligence becomes an attractive option, with deep learning as a promising tool for pattern recognition and outcome prediction [13, 14]. Deep learning uncovers complex structures in large datasets by using the backpropagation algorithm to indicate how an algorithm should adjust its internal parameters that are used to compute the representation in each layer from the representation in the previous layer [14]. Deep learning-based techniques have been already shown to provide an accuracy advantage in bladder cancer recurrence and progression prediction [15]. Artificial neural network (ANN) constitutes one of the most commonly used methods of deep learning and provides many benefits (e.g. ability to implicitly detect complex nonlinear relationships between dependent and independent variables) but it is not devoid of limitations (e.g. the ‘black-box’ problem) [16]. One of the studies showed the accuracy benefit of ANN compared to the CPH model in the prediction of CSS, but not OS in bladder cancer patients treated with cystectomy [17]. There is a particular necessity to estimate the risk of progression and cancer-specific death in T1 BC to qualify patients, who are at risk, for more radical treatment—early RC [2]. This unmet need constituted the rationale to focus on mortality prediction in this particular group of bladder cancer patients, in whom therapeutic decisions are especially challenging. On the other hand, overall- and other-cause mortality is another outcome requiring attention to facilitate the selection of patients for more conservative management and thus avoiding overtreatment.

In this population-based study, we aimed to identify factors associated with OS and CSS in patients with T1 NMIBC treated with TURBT. Furthermore, we aimed to compare the utility of ANN and conventional machine learning tools – LR in the prediction of 5-year CSS and OS in patients with T1 NMIBC.

## Methods

The Surveillance, Epidemiology, and End Results (SEER) database (2004–2015) which consists of seventeen different registries, lastly updated in November 2021 was used for this research. The SEER*Stat statistical software was used to select patients meeting the study criteria. A systematic search was performed to identify patients, who were diagnosed with T1 NMIBC between 2004 and 2015, in accordance with the 6^Th^ Derived American Joint Committee on Cancer (AJCC) T edition (ICD-10 codes from C67.0 to C67.9 with the exclusion of C67.7—urachal tumour). In the further selection, only patients treated with TURBT were included in the analysis. We excluded patients with evidence of nodal involvement or distant metastases, with unknown metastatic status due to lack of imaging studies, previous radiotherapy for BC, previous evidence of muscle-invasive BC, missing tumour characteristics (e.g. grading), and unknown survival status.

The available data included information on the patient’s sociodemographics, tumour histopathological (e.g. T category, grading) and clinical characteristics (e.g. tumour size, location, history of the previous tumour defined as non-primary bladder malignancy), a therapy used (e.g. surgery type, chemotherapy) and survival outcomes (cancer and all-cause mortality during the follow-up). The 5-year OS and CSS was calculated for patients with sufficient follow-up as previously reported [18, 19].

The dataset was divided into training and validation cohorts in the proportion of 70 to 30. Group characteristics are presented in the respective table with the number of patients and percentage for categorical variables and medians, accompanied by the interquartile range (IQR) for continuous variables.

### Statistical analysis

Statistical analyses were conducted in SAS software (Cary, NC, U.S.) version 9.4 and using R version 4.2.1 (R Foundation for Statistical Computing, Vienna, Austria). Multivariable analyses using LR and CPH with stepwise selection of variables were performed in SAS software. Odds ratio (OR) and hazard ratio (HR) supplemented with a 95% confidence interval (95% CI) were derived from multivariable regression. For all statistical analyses, we considered a two-sided p value < 0.05 as statistically significant.

Artificial neural networks were developed using R language and ‘neuralnet’ package. A resilient backpropagation algorithm (‘Rprop + ’) was utilized with one layer and four hidden neurons (vertices) to predict 5-year survival outcomes. Only factors that were significantly associated with mortality in multivariable analyses with LR were used as input variables to build a neural network. Cross-entropy was used as a differentiable function for the calculation of the error. The threshold specifying the partial derivatives of the error function as stopping criteria in neural network creation was set as 0.1. Each learning process for ANN included two repetitions.

Separate predictive models for 5-year OS and 5-year CSS were developed using both LR and ANN in the training cohort. Then, all models were tested in the validation cohort. The area under the curve (AUC) for receiver operating characteristic (ROC) and model accuracy derived from the confusion matrix were calculated. Accuracy was defined as the proportion of correct predictions to all predictions.

## Results

### Clinicopathologic features

Overall 32,060 patients with T1 bladder cancer who underwent TURBT were included in the analysis, 22,442 patients were subjected to the training cohort and 9618 patients to the validation one respectively. Training and validation cohorts did not differ in the frequency of characteristics and outcomes (Table 1). The majority of patients were males (N = 25,049; 78%), of the white race (N = 28,587; 89%) and over 70 years of age (N = 19,955; 62%). High-grade tumours (N = 22,115; 69%), mostly of urothelial histology (N = 31,265; 97.5%) were predominant. The majority of tumours were located in the anterior/posterior or lateral bladder walls (N = 12,020; 37.5%) or the location was not specified (e.g. multiple location) (N = 13,722; 42.8%), less frequently tumours originated from the bladder neck or trigone (N = 2802; 8.7%), or extended through more than one area of the bladder (N = 3516; 11%). The vast majority of patients presented with primary T1 bladder tumour (N = 29,055; 90.6%) and 9.4% had a previous history of NMIBC.

**Table 1:** Clinicopathologic and sociodemographic characteristics of patients with T1 bladder cancer treated with transurethral resection of the bladder tumour for the whole cohort, training and validation groups

Characteristics	Reference	Whole cohort		Training cohort		Validation cohort	
		Number of pts	%	Number of pts	%	Number of pts	%
Gender	Male	25,049	78.13	17,515	78.05	7534	78.33
	Female	7011	21.87	4927	21.95	2084	21.67
Age (years)	< 60	4460	13.91	3139	13.99	1321	13.73
	60–70	7645	23.85	5348	23.83	2297	23.88
	70–80	10,004	31.2	7014	31.25	2990	31.09
	> 80	9951	31.04	6941	30.93	3010	31.30
Tumour grading	Low-grade	9945	31.02	6943	30.94	3002	31.21
	High-grade	22,115	68.98	15,499	69.06	6616	68.79
Tumour histology	Urothelial	31,265	97.52	21,894	97.56	9371	97.43
	Squamous	307	0.96	205	0.91	102	1.06
	Other	488	1.52	343	1.53	145	1.51
Tumour location	Trigone/neck	2802	8.74	1981	8.83	821	8.54
	Lateral/anterior/posterior	12,020	37.49	8386	37.37	3634	37.78
	More than one area	3516	10.97	2470	11.01	1046	10.88
	Not specified/ multiple	13,722	42.8	9605	42.80	4117	42.81
History of previous NMIBC	Yes	3005	9.37	2100	9.36	905	9.41
	No	29,055	90.63	20,342	90.64	8713	90.59
Previous tumour stage	Ta	1497	49.82	1048	49.90	449	49.61
	CIS	209	6.96	138	6.57	71	7.85
	Unknown stage	1299	43.23	914	43.52	385	42.54
Tumour size	< 3 cm	5721	17.84	4049	18.04	1672	17.38
	≥ 3 cm	8742	27.26	6160	27.45	2582	26.85
	Unknown	17,597	54.89	12,233	54.51	5364	55.77
Race	White	28,587	89.17	20,014	89.18	8573	89.13
	Black	1706	5.32	1200	5.35	506	5.26
	Other*	1767	5.51	1228	5.47	539	5.60
Marital status	Unmarried	10,847	33.83	7576	33.76	3271	34.01
	Married **	19,079	59.51	13,343	59.46	5736	59.64
	Unknown	2134	6.66	1523	6.79	611	6.35
Income annually	< $65,000	14,110	44.01	9898	44.10	4212	43.79
	≥ $65,000	17,950	55.99	12,544	55.90	5406	56.21
Presence of other malignancy	Yes	8264	25.78	5806	25.87	2458	25.56
	No	23,796	74.22	16,636	74.13	7160	74.44
Metropolitan citizenship***	No	3806	11.87	2645	11.79	1161	12.07
	Yes	28,254	88.13	19,797	88.21	8457	87.93
Survival (months)	Median/IQR	116	80–153	116	80–153	116	81–153
Cancer-related death	No	24,830	77.45	17,391	77.49	7439	77.34
	Yes	7230	22.55	5051	22.51	2179	22.66
Death	No	13,575	42.34	9498	42.32	4077	42.39
	Yes	18,485	57.66	12,944	57.68	5541	57.61
5-year cancer-specific mortality	No	17,382	54.22	12,172	54.24	5210	54.17
	Yes	5650	17.62	3956	17.63	1694	17.61
	n/a or unknown****	9028	28.16	6314	28.13	2714	28.22
5-year overall mortality	No	17,382	54.22	12,172	54.24	5210	54.17
	Yes	12,522	39.06	8736	38.93	3786	39.36
	n/a or unknown****	2156	6.72	1534	6.84	622	6.47

*Includes American Indian/Alaska Native/Asian or Pacific Islander

**Partnership without official marriage was also regarded as married status

***Citizenship of counties in metropolitan area

****Regarded as no event in the available follow-up, only for follow-up shorter than 5 years

### Survival outcomes

Cancer-specific deaths occurred in 22.6% of cases (N = 7230), whereas all-cause mortality exceeded 57.6% (N = 18,485) during the median follow-up of 116 months (IQR 80–153 months). The 5-year OS exceeded 58%. Noteworthy, 9028 patients, who were excluded from the prediction of 5-year CSS included those who were lost to follow-up (N = 2156; 6.7%) and those who died of other causes before 5 years of follow-up (cardiovascular diseases—2658 pts/ 8.3%; other cancer—1563 pts/4.9%; lung diseases and infections 759/2.3% and other mortality causes 1868 pts/5.8%). These exclusions were made due to competing event in the majority of cases and lost to follow-up in the minority. Comparison of included and excluded patients’ characteristics are shown in the supplementary table 1.

### CSS prediction with LR and ANN

Multivariable analyses with LR were performed and revealed tumour grade (high-grade vs low-grade; HR = 2.07 95% CI 1.88–2.29 p < 0.001), size (≥ 3 cm vs < 3 cm; HR = 1.36 95% CI 1.19–1.56 p < 0.05), histology variant (squamous vs urothelial; HR = 5.56 95% CI 3.88–7.96 p < 0.001; other vs urothelial; HR = 3.38 95% CI 2.53–4.51 p < 0.001), location (lateral/posterior/anterior wall vs trigone/neck; HR = 0.78 95% CI 0.66–0.91 p < 0.001; more than one bladder area vs trigone/neck; HR = 1.20 95% CI 1.00–1.44 p < 0.001; not specified/multiple tumour location vs trigone/neck HR = 1.11 95% CI 0.95–1.44 p < 0.01), primary tumour character (recurrent vs primary; HR = 1.60 95% CI 1.40–1.83 p < 0.001), patient’s age (> 80 yr vs < 60 yr; HR = 7.35 95% CI 6.29–8.60 p < 0.001; 70–80 yr vs < 60 yr; HR = 2.50 95% CI 2.13–2.93 p < 0.05; 60–70 yr vs < 60 yr; HR = 1.48 95% CI 1.25–1.76 p < 0.01), race (black vs white; HR = 1.58 95% CI 1.33–1.88 p < 0.001; other vs white; HR = 0.83 95% CI 0.68–1.00 p < 0.001), marital status (married vs unmarried; HR = 0.68 95% CI 0.63–0.75 p < 0.001) and annual income (higher vs lower; HR = 0.90 95% CI 0.83–0.98 p < 0.05) as factors predicting 5-year CSS (Table 2). The discrimination of the LR-based model reflected by the area under the ROC curve was 73.4%, whereas the accuracy of prediction exceeded 79.5% in the validation cohort. Using the same variables ANN was created and achieved the same AUC and accuracy as LR in the validation cohort (72.5% and 79.4% respectively). Moreover, for both, LR and ANN predictions’ accuracy and AUC were comparable in the training and validation cohorts, which pinpoints the small risk of overfitting. Multivariable analyses performed using CPH and LR revealed the same risk factors for CSS (supplementary table 2).

**Table 2:** Factors predicting 5-year cancer-specific survival of patients with T1 bladder cancer treated with transurethral resection of the bladder tumour

Factors predicting 5-year cancer-specific survival- multivariable analysis				
Variables	Reference	OR	95% CI	P value
Age (years)	< 60	Ref	–	< 0.0001
	60–70	1.486	1.253–1.762	< 0.0001
	70–80	2.496	2.127–2.930	0.0163
	> 80	7.354	6.285–8.604	< 0.0001
Tumour grading	High- vs low-grade	2.073	1.876–2.289	< 0.0001
Tumour size	< 3 cm	Ref	–	< 0.0001
	≥ 3 cm	1.361	1.185–1.563	0.0437
	Unknown	1.498	1.321–1.698	< 0.0001
Tumour histology	Urothelial	Ref	–	< 0.0001
	Squamous	5.557	3.878–7.963	< 0.0001
	Other variants	3.381	2.534–4.512	0.0355
Tumour location	Trigone/neck	Ref	–	< 0.0001
	Lateral/anterior/posterior	0.778	0.664–0.910	< 0.0001
	More than one area	1.196	0.995–1.438	0.0008
	Not specified/ multiple	1.111	0.953–1.294	0.0057
History of previous NMIBC	Yes vs no	1.599	1.399–1.829	< 0.0001
Marital status	Unmarried	Ref	–	< 0.0001
	Married*	0.683	0.625–0.747	0.0003
	Unkown	0.689	0.578–0.821	0.0323
Income annually	≥ $65,000 vs < $65,000	0.902	0.829–0.982	0.0177
Race	White	Ref	–	< 0.0001
	Black	1.577	1.325–1.876	< 0.0001
	Other**	0.825	0.679–1.001	< 0.0001

*OR* odds ratio, *CI* confidence interval, *ref*. reference

*Partnership without official marriage was also regarded as married status

**Includes American Indian/Alaska Native/Asian or Pacific Islander

### OS prediction with LR and ANN

The following factors were significant predictors of 5-year OS: tumour grade, size, histology variant, location, patient’s age, race and gender, presence of other malignancy, marital status, annual income, and metropolitan citizenship (Table 3). AUC for the LR and ANN models were 74.3% and 74.2% respectively in the training cohort. Similarly, in the validation cohort, the LR-based model and ANN achieved the same AUC (73.7% vs 73.4% respectively) and same accuracy (69.1% vs 69.3% respectively) for the prediction of 5-year OS. Summarized values of accuracy and AUC for both ANN and LR-based models predicting CSS and OS are presented in the Table 4.

**Table 3:** Factors predicting 5-year overall survival of patients with T1 bladder cancer treated with transurethral resection of the bladder tumour

Factors predicting 5-year overall survival- multivariable analysis				
Variables	Reference	OR	95% CI	P value
Gender	Female vs male	0.814	0.754–0.879	< 0.0001
Age (years)	< 60	Ref	–	< 0.0001
	60–70	1.713	1.515–1.936	< 0.0001
	70–80	3.471	3.094–3.895	< 0.0001
	> 80	9.787	8.712–0.996	< 0.0001
Tumour grading	High- vs low-grade	1.471	1.377–1.573	< 0.0001
Tumour size	< 3 cm	Ref	–	< 0.0001
	≥ 3 cm	1.213	1.104–1.333	0.0274
	Unknown	1.251	1.149–1.362	< 0.0001
Tumour histology	Urothelial	Ref	–	< 0.0001
	Squamous	3.301	2.391–4.557	< 0.0001
	Other	2.128	1.666–2.719	0.2867
Tumour location	Trigone/neck	Ref	–	< 0.0001
	Lateral/anterior/posterior	0.872	0.779–0.976	< 0.0001
	More than one area	1.152	1.006–1.320	0.0007
	Not specified/ multiple	1.049	0.939–1.172	0.1779
Presence of other malignancy	Yes vs no	1.506	1.406–1.613	< 0.0001
Marital status	Unmarried	Ref	–	< 0.0001
	Married*	0.685	0.640–0.733	< 0.0001
	Status unkown	0.719	0.632–0.818	0.0243
Income annually	≥ $65,000 vs < $65,000	0.894	0.838–0.954	0.0007
Race	White	Ref	–	< 0.0001
	Black	1.339	1.170–1.533	< 0.0001
	Other**	0.799	0.694–0.919	< 0.0001
Metropolitan citizenship***	Yes vs no	0.885	0.801–0.977	0.0151

*OR* odds ratio, *CI* confidence interval, *ref*. reference

*Partnership without official marriage was also regarded as married status

**Includes American Indian/Alaska Native/Asian or Pacific Islander

***Citizenship of counties in metropolitan area

**Table 4:** Accuracy and area under the curve for artificial neural network (ANN) and logistic regression (LR) based models predicting 5-year cancer-specific (CSS) and overall survival (OS) in the training and validation cohorts

		CSS		OS	
		LR	ANN	LR	ANN
Training cohort	Accuracy (%)	80.1	79.8	68.2	68.8
	AUC (%)	74.3	74.1	74.3	74.2
Validation cohort	Accuracy (%)	79.5	79.4	69.1	69.3
	AUC (%)	73.4	72.5	73.7	73.4

## Discussion

In this population-based study involving 32 060 patients with T1 bladder cancer, we aimed to identify predictive factors for survival and compare the accuracy of 5-year CSS and OS predictions with the use of ANN and LR-based models. Firstly and most importantly, multiple factors are associated with unfavourable CSS including clinical and histopathological features: older age, higher histological grade, non-urothelial histology variants, tumour size ≥ 3 cm, recurrent tumour, tumour location, and sociodemographic features such as black race, non-married status and lower annual income.

Secondly, the analysis showed that ANN is a feasible prediction tool with acceptable, moderate accuracy in survival estimation. Thirdly, ANN and conventional machine learning method—LR-based model exhibit the same discrimination and prediction accuracy for 5-year CSS and OS in T1 NMIBC. Forth, pursuit for better prognostic tools in T1 BC is of great significance as the reported real-world survival outcomes remain poor.

The above results raise the question if the neural network is a valid approach for survival model creation. The accuracy was no better than the one of the conventional LR models. Moreover, identical prediction factors as for LR were provided for ANN and yielded the same accuracy. The obvious drawback of the ANN is the ‘black-box’ problem, which means that we cannot exactly know which factors play the most significant role and how the ANN learning process goes on. However, as ANN seem to be not inferior to LR it must be considered a valid approach to be used in future studies and web applications for clinical use. Perhaps the optimization (e.g. hyperparameters modification) might be a clue to the success and ANN models could become a valuable tool for clinical implementation. Disadvantages coming from the so-called ‘black-box’ problem are also possible to overcome as suggested by some authors [20]. Moreover, such black-box algorithms can allow the health system to leverage complex biological relationships well before those relationships are understood [21].

Artificial intelligence with the underscored role of deep-learning techniques such as neural networks has an undeniable role to play in current and future medicine [22]. It can assist pathologists and radiologists in image analysis or even replace specialists in some cases and spare human healthcare resources [22]. Current studies show very promising accuracy of image recognition, even more accurate than the assessment performed by physicians [23]. The benefits of neural networks can be also harnessed in the field of oncology for molecular studies, pharmacogenomics, microscopic assessment and prognosis prediction [24].

Widely used EORTC and CUETO risk scores or EAU risk groups were not designed to estimate the risk of cancer-specific or overall mortality. Above classifications were established to stratify patients according to the progression- and/or recurrence-free survival, but not CSS [4, 11, 12]. The studies predicting CSS in high-risk NMIBC, including the group of T1 bladder tumours are lacking. In our analysis, cancer-specific deaths were recorded in 22.5% of patients within a median follow-up of 166 months (IQR 80–153). A systematic review of prospective trials including 1183 high-risk NMIBC patients (mainly T1 stage) reported an average of 15% of CSM within shorter follow-up (median from 52 to 123 months, ranging between studies) [25]. Thus, we argue that due to its aggressiveness, T1 NMIBC should be also regarded as potentially deadly and risks of both progression and cancer-specific death should be estimated to aid clinical decision-making.

We identified clinical, histopathological and sociodemographic factors that were independently associated with CSS. These included obvious factors such as high-grade tumour, history of previous NMIBC and non-urothelial histology variants. High-grade histology is the strongest risk factor for progression in NMIBC [26] and is most commonly reported for T1 tumours, which are almost always considered ‘high-risk’ as they infiltrate the submucosa of the bladder [27]. To date, the significance and prognosis of low-grade T1 tumours were enigmatic. However, recent multicenter retrospective studies showed that T1G1 are characterized by a significantly higher progression rate than TaG1 tumours [27] and T1LG conferred a better prognosis than T1HG [28]. Nonetheless, our analysis supports the rationale for cautious and precise grading assessment in T1 tumours (although being always ‘high-risk’, T1 is not always high-grade). Recognition of low-grade T1 is possible, necessary and rationalized by the distinct prognosis of T1 low-grade and high-grade tumours, which is also confirmed in our study. The history of previous NMIBC is a known risk factor for further recurrences and progression in T1, which was already mentioned in other studies [4, 11]. Finally, non-urothelial histology of BC confer a risk factor for progression [29]. Pure SCC is not responsive to BCG and characterized by higher progression rates and should be a priori treated with RC [30]. Therefore, T1 patients with SCC and other than pure urothelial variants of histology should be counselled on high progression risk following sole TURBT and informed on the benefits of RC. Other factors such as older age, large tumour size and multiple locations of the tumour are also underlined as predictors of progression in multiple studies [3, 4, 11, 12]. Especially challenging is the therapeutic strategy in elderly patients who on the one hand are at high risk for complications after cystectomy, but on the other hand, are at highest risk of progression when treated with bladder-sparing approach [12, 31, 32]. Perhaps optimization of adjuvant intravesical therapies is most desirable to prevent progression in elderlies, who are unfit for RC. Important, but tremendous conclusions come from the observation that sociodemographic features influence the CSS. The relationship between the lower than median annual income and worse CSS indicates the disparity in access to healthcare and treatment. Black race was shown to confer another risk factors which might result from genetic dependencies or again unequal access to healthcare providers [33]. The issue of being married as a protective factor has been already shown in other population-based studies in urothelial cancer [34, 35] and this might underline the role of supportive relatives and partners in the process of treatment and strict follow-up.

Factors influencing the OS were similar to those predicting CSS. The following factors were associated with worse OS: high-grade tumour, tumour size ≥ 3 cm, non-urothelial histology, tumour location, older age, black race, male gender, the presence of other malignancy, non-married status, lower annual income and non-metropolitan citizenship. Tumour-related factors such as high-grade histology, variants of histology, and large size contributed to worse OS, most probably due to their association with higher CSM. Older age and the presence of other malignancy increase the risk of all-cause mortality. The male gender was associated with worse OS, which supports the general rule of shorter life expectancy in men. Conversely, consistently other authors show poorer bladder cancer-specific survival in women than in men [36]. Importantly, non-metropolitan citizenship was related to worse survival and this might be attributed to worse accessibility to medical care in rural areas [37]. The association between the development of other primary malignancy and worse OS, underscores the need for other cancer awareness and the necessity to provide holistic care to the patient. Concomitant malignancies in patients with BC seem to originate most often from the prostate or the upper urinary tract [38, 39].

Limitations of the study include the lack of detailed data on the patient comorbidities, smoking habits, and some tumour characteristics (e.g. lymphovascular invasion) and most importantly the quality of surgery (e.g. detrusor muscle in the specimen, macroscopically radical resection) which all have been proven to influence the prognosis [2, 40]. The prediction of OS was mainly limited by the lack of the data on comorbidities. On the other hand, the use of sociodemographic factors (e.g. race, income) might not be reliable prognostic factors in everyday clinical practice. The information about the adjuvant use of intravesical therapies (e.g. BCG) was unreliable in most cases with only a few reports of BCG use despite clear indication for BCG in the majority of T1 BC patients. We might suppose that many patients received BCG, but it was not recorded in the SEER database and this must be considered an important, uncontrolled confounder. Survival outcomes were available for the majority of patients with acceptable median follow-up after surgery, but no relevant information about progression or recurrence could be received. Moreover, in our analysis we did not validate the reliability of EORTC risk tables in CSS prediction. Some variables, including the presence of concomitant carcinoma in situ, were not available, whereas the data on tumour multiplicity was inaccurate. The data about primary character of the tumour and its location in the bladder (according to ICD-10 coding) was available for all patients. Exact tumour diameter was available for the 45% of analysed patients and remaining 55% of patients were assigned as of unknown tumour size (Table 1). Above risk factors contributing to EORTC risk score calculation were therefore not fully available.

Analysis of 5-year OS and CSS required the exclusion of patients with insufficient follow-up which must be regarded as bias. Since both cancer-specific and overall mortality are time-dependent variables, analysing them as binary end-point might raise some methodological concerns and it should be clearly reported as a limitation. We have, however, performed consecutive analyses to confirm the utility of such an approach. Performed CPH analysis yielded the same predictors for CSS as LR. Among patients excluded from survival prediction, only 6.7% were lost to follow-up before 60 months. Other patients which were excluded from CSS analysis, died from other diseases than BC as reported in the results section. The bias of excluding the patients who did not reach the 5-year follow-up was limited by the performed comparison between the analyzed and excluded patients (supplementary table 1). The only main clinically significant difference between included and excluded patients was more advanced age in the latter ones contributing to higher other-cause mortality (supplementary table 1). A binary endpoint (5-year CSM) seems therefore acceptable for the comparison of LR and ANN performance. The accuracy of prediction in the training and validation datasets was very similar which indicates a rather small risk of model overfitting.

To conclude, available predictive factors for CSS and OS might be useful to estimate the risk of 5-year CSS and OS in patients with T1 BC. The accuracy of mortality prediction is still moderate, also when ANN is applied. Patients with T1 BC and adverse features should receive more aggressive treatment after initial TURBT.

## Supplementary Information

Below is the link to the electronic supplementary material.

Supplementary file1 (DOCX 23 KB)

## Data Availability

The datasets analysed during the current study are available from the US National Cancer Institute upon request.
