# Supplementary material for: The prediction of cancer-specific mortality in T1 non-muscle-invasive bladder cancer: comparison of logistic regression and artificial neural network: a SEER population-based study
Source: Int Urol Nephrol. 2023 Jun 6;55(9):2205–13. doi: 10.1007/s11255-023-03655-5 (PMC10406653; doi:10.1007/s11255-023-03655-5)
Supplement: Supplementary file 1 — Supplementary file1 (DOCX 23 KB) [file 11255_2023_3655_MOESM1_ESM.docx]

**Supplementary file 1**

**Table.1. Comparison of clinicopathologic features between patients included in the analysis of cancer-specific survival and excluded due to insufficient follow-up.**

|  |  | Included | | Excluded | |  |
| --- | --- | --- | --- | --- | --- | --- |
| **Characteristics** | Ref. | Number of pts. | % | Number of pts. | % | P |
| Gender | male | 17832 | 77.42 | 7217 | 79.94 | <.0001* |
|  | female | 5200 | 22.58 | 1811 | 20.06 |  |
| Age (years) | <60 | 3742 | 16.25 | 718 | 7.95 | <.0001* |
|  | 60-70 | 6126 | 26.60 | 1519 | 16.83 |  |
|  | 70-80 | 7189 | 31.21 | 2815 | 31.18 |  |
|  | >80 | 5975 | 25.94 | 3976 | 44.04 |  |
| Tumour grading | low-grade | 7304 | 31.71 | 2641 | 29.25 | <.0001* |
|  | high-grade | 15728 | 68.29 | 6387 | 70.75 |  |
| Tumour histology | urothelial | 22458 | 97.51 | 8807 | 97.55 | 0.6766 |
|  | squamous | 227 | 0.99 | 80 | 0.89 |  |
|  | other | 347 | 1.51 | 141 | 1.56 |  |
| Tumour location | trigone/neck | 2042 | 7.87 | 760 | 8.42 | 0.28 |
|  | lateral/anterior/posterior | 8607 | 37.37 | 3413 | 37.80 |  |
|  | more than one area | 2492 | 10.82 | 1024 | 11.34 |  |
|  | not specified/ multiple | 9891 | 42.94 | 3831 | 42.43 |  |
| Tumour size | < 3 cm | 4109 | 17.84 | 1612 | 17.86 | 0.0029* |
|  | ≥ 3 cm | 6163 | 26.76 | 2579 | 28.57 |  |
|  | unknown | 12760 | 55.40 | 4837 | 53.58 |  |
| Race | white | 20499 | 89.00 | 8088 | 89.59 | 0.2808 |
|  | black | 1251 | 5.43 | 455 | 4.05 |  |
|  | other* | 1282 | 5.57 | 485 | 5.37 |  |

*The differences between the groups of included and excluded patients were statistically significant but not clinically significant for gender (22% vs 20% of females), tumour grade (68% vs 70.8% of high-grade histology), tumour size > 3 cm (28.6% vs 26.7%). Only, the age of patients seems to significantly differ between included and excluded patients.

**Table.2. Multivariable model predicting cancer-specific survival using Cox proportional hazards in patients with T1 bladder cancer.**

| Factors predicting cancer-specific survival - multivariable analysis | | | | |
| --- | --- | --- | --- | --- |
| Variables | Reference | HR | 95% CI | P-value |
| Age (years) | <60 | ref. | - | <.0001 |
|  | 60-70 | 1.401 | 1.253-1.566 | <.0001 |
|  | 70-80 | 2.186 | 1.970-2.427 | <.0001 |
|  | >80 | 5.131 | 4.638-5.676 | <.0001 |
| Tumour grading | high- vs low-grade | 1.640 | 1.542-1.745 | <.0001 |
| Tumour size | < 3 cm | ref. |  | <.0001 |
|  | ≥ 3 cm | 1.330 | 1.218-1.453 | <.0001 |
|  | unknown | 1.413 | 1.304-1.530 | <.0001 |
| Tumour histology | urothelial | ref. |  | <.0001 |
|  | squamous | 3.418 | 2.847-4.104 | <.0001 |
|  | other variants | 2.381 | 2.022-2.804 | <.0001 |
| Tumour location | trigone/neck | ref. |  | <.0001 |
|  | lateral/anterior/posterior | 0.855 | 0.774-0.943 | 0.0018 |
|  | more than one area | 1.190 | 1.062-1.333 | 0.0027 |
|  | not specified/ multiple | 1.096 | 0.997-1.206 | 0.0586 |
| History of previous NMIBC | yes vs no | 1.443 | 1.334-1.561 | <.0001 |
| Marital status | unmarried | ref. |  | <.0001 |
|  | married | 0.729 | 0.690-0.771 | <.0001 |
|  | status unkown | 0.734 | 0.656-0.821 | <.0001 |
| Income annually | ≥ $65,000 vs < $65,000 | 0.903 | 0.857-0.952 | 0.0002 |
| Race | white | ref. |  | <.0001 |
|  | black | 1.375 | 1.237-1.530 | <.0001 |
|  | other | 0.870 | 0.769-0.983 | 0.0254 |
